# Combining protein and metabolic engineering strategies for biosynthesis of melatonin in *Escherichia coli*

**DOI:** 10.1186/s12934-021-01662-8

**Published:** 2021-08-28

**Authors:** Yanfeng Zhang, Yongzhi He, Nan Zhang, JiaJia Gan, Shan Zhang, Zhiyang Dong

**Affiliations:** 1 State Key Laboratory of Microbial Resources, Institute of Microbiology, Chinese Academy of Sciences, No. 1 West Beichen Road, Chaoyang District, Beijing, 100101 People’s Republic of China; 2 University of Chinese Academy of Sciences, Beijing, 100049 People’s Republic of China; 3 Shenzhen Siyomicro Bio-Tech C., LTD, No. 39 Qingfeng Avenue, Baolong Community, Longgang District, Shenzhen, 518116 People’s Republic of China

**Keywords:** Melatonin, *N*-acetylserotonin, Metabolic engineering, *Streptomyces albulus*, *S*-adenosylmethionine

## Abstract

**Background:**

Melatonin has attracted substantial attention because of its excellent prospects for both medical applications and crop improvement. The microbial production of melatonin is a safer and more promising alternative to chemical synthesis approaches. Researchers have failed to produce high yields of melatonin in common heterologous hosts due to either the insolubility or low enzyme activity of proteins encoded by gene clusters related to melatonin biosynthesis.

**Results:**

Here, a combinatorial gene pathway for melatonin production was successfully established in *Escherichia coli* by combining the physostigmine biosynthetic genes from *Streptomyces albulus* and gene encoding phenylalanine 4-hydroxylase (P4H) from *Xanthomonas campestris* and caffeic acid 3-*O*-methyltransferase (COMT) from *Oryza sativa*. A threefold improvement of melatonin production was achieved by balancing the expression of heterologous proteins and adding 3% glycerol. Further protein engineering and metabolic engineering were conducted to improve the conversion of *N*-acetylserotonin (NAS) to melatonin. Construction of COMT variant containing C303F and V321T mutations increased the production of melatonin by fivefold. Moreover, the deletion of *speD* gene increased the supply of S-adenosylmethionine (SAM), an indispensable cofactor of COMT, which doubled the yield of melatonin. In the final engineered strain EcMEL8, the production of NAS and melatonin reached 879.38 ± 71.42 mg/L and 136.17 ± 1.33 mg/L in a shake flask. Finally, in a 2-L bioreactor, EcMEL8 produced 1.06 ± 0.07 g/L NAS and 0.65 ± 0.11 g/L melatonin with tryptophan supplementation.

**Conclusions:**

This study established a novel combinatorial pathway for melatonin biosynthesis in *E. coli* and provided alternative strategies for improvement of melatonin production.

**Supplementary Information:**

The online version contains supplementary material available at 10.1186/s12934-021-01662-8.

## Background

Melatonin is an ancient and ubiquitous molecule [1], widely distributed in almost all taxa of living organisms, including microorganisms, plants, and animals [2]. Melatonin is considered one of nature’s most versatile biological signals, and its functions have diverged with organismal diversification [3]. Due to its multiple functions [4], melatonin has shown excellent prospects in both medical applications and crop improvement [5–7]. Melatonin has been extensively used as an over-the-counter drug and a dietary supplement for many years word wide. Currently, commercial melatonin relies on chemical synthesis, which is neither sustainable nor environmentally friendly [8–10]. The microbial production of melatonin is a safer and more promising alternative based on the understanding of the melatonin biosynthetic pathway [9].

The biosynthetic pathway of melatonin was first elucidated in animals [11]. In animals, melatonin is synthesized from tryptophan via the 5-hydroxytryptophan (5HTP), serotonin (5HT), and *N*-acetylserotonin (NAS) intermediates. Accordingly, four enzymes, including tryptophan-5-hydroxylase (TPH), tryptophan decarboxylase (TDC), serotonin *N*-acetyltransferase (SNAT), and *N*-acetylserotonin methyltransferase (ASMT), are involved in the catalytic process [11]. In plants, melatonin biosynthesis also begins with tryptophan and includes four enzymatic steps. However, the process of melatonin biosynthesis in plants differs from that in animals in several aspects [12]. First, the first enzymatic step is tryptophan decarboxylation rather than hydroxylation, as occurs in animals [13, 14]. Second, the subsequent step is the synthesis of 5-HT, which catalyzed by tryptamine 5-hydroxylase (T5H) [15]. Third, in addition to ASMT, caffeic acid O-methyltransferase (COMT), which is absent in animals, is another enzyme involved in the synthesis of melatonin. It is worth mentioning that COMT has a significantly higher catalytic efficiency than ASMT in the conversion of *N*-acetylserotonin to melatonin. The catalytic efficiency (*V*_*max*_*/K*_*m*_) for COMT activity was 709-fold higher than for ASMT in *Arabidopsis thaliana*, indicating a pivotal role of COMT in the synthesis of melatonin [16]. Therefore, in plants, a total of six enzymes, namely, TPH, SNAT, ASMT, TDC, T5H, and COMT, are involved in the biosynthetic pathway of melatonin, suggesting the complexity of melatonin biosynthesis [12]. In addition to the classic pathway, an alternate pathway, in which serotonin is first *O*-methylated and the resulting 5-MT is *N*-acetylated, was recently proposed, adding further complexity to the pathway of melatonin biosynthesis [12, 17]. Compared with the number of studies in animals and plants, there are few studies on the biosynthetic pathway in microorganisms, although melatonin is believed to have first appeared in bacteria as early as billions of years ago [3, 17]. The enzymes and corresponding genes involved in melatonin biosynthesis in microorganisms have remained almost unknown.

Therefore, almost all the genes necessary to establish the melatonin biosynthetic pathway in genetically engineered bacteria were cloned from animals and plants [18–20]. Germann et al. [19] established a de novo melatonin biosynthetic pathway in recombinant yeast. In that work, the genes encoding TPH were from either *Homo sapiens* or *Schistosoma mansoni.* Moreover, the genes encoding TDC and ASMT were cloned from *H. sapiens,* and the gene encoding SNAT was cloned from *Bos taurus.* Recently, Luo et al. established a recombinant melatonin biosynthetic pathway in *E. coli.* The genes encoding TPH and ASMT were cloned from *H. sapiens* and the genes encoding TDC and SNAT were cloned from *Candidatus Koribacter versatilis* and *Streptomyces griseofuscus* respectively [21]. In other attempts to produce melatonin in *E. coli*, genes encoding SNAT and ASMT were cloned from several animals and plants. When these enzymes derived from mammals and plants are heterologous expressed in *E. coli*, the expression level is extremely low or the expression product is inactive, which limits the high production of melatonin in prokaryotic cells [20, 22].

To date, the gene cluster involved in melatonin biosynthesis in microorganisms has not been identified. However, a gene cluster responsible for physostigmine biosynthesis in *Streptomyces* harbors the first three genes that encode enzymes to produce NAS, which is the immediate precursor of melatonin [23]. Physostigmine, a tryptophan-derived heterocyclic alkaloid is first discovered in the seeds of West African beans. As a potent acetylcholine inhibitor, physostigmine is widely used to treat glaucoma and Alzheimer’s disease but there are few studies regarding its in-situ function and biosynthesis pathway [24, 25]. After large scale-up screening, the researchers discovered that submerged cultivation of the actinomycetes *Streptomyces griseofuscus* and *Streptomyces pseudogriseolus* can produce physostigmine [25]. In 2014, Liu et al. [23] identified the gene cluster *psmA-H* responsible for the biosynthesis of physostigmine in *Streptomyces griseofuscus*. Of these, *PsmH* and *PsmF* are required for the NAS biosynthesis starting from 5-HTP, which is shared with the melatonin pathway. Based on the physostigmine biosynthetic pathway, in this paper, a novel biosynthetic pathway for melatonin production was established in *E. coli*. In addition, metabolic engineering and enzyme engineering strategies were employed to optimize the biosynthetic pathway and the production of melatonin was effectively improved by 11-fold compared to the first generation strain.

## Materials and methods

### Bacterial strains and media

All bacterial strains used in this study are listed in Table 1. *E. coli* Trans1-T1 (TransGen, Beijing, China) was used as the host strain for plasmid construction and propagation. *E. coli* BW25113*∆tnaA* was used for protein expression and in vivo hydroxylation of L-tryptophan to melatonin. For efficient biosynthesis of melatonin, genomic modification of the *E. coli* BW25113*∆tnaA* strain was performed using λ-red recombination [26]. Luria–Bertani (LB) medium containing 10 g/L tryptone, 5 g/L yeast and 10 g/L NaCl was used for cell cultivation and enzyme expression. Modified M9 medium (M9Y) was used for the in vivo production of 5-HTP and melatonin from L-tryptophan in shake flasks [22]. M9Y medium contained 10 g/L glucose, 2 g/L yeast extract, 6 g/L Na_2_HPO_4_, 0.5 g/L NaCl, 1 g/L NH_4_Cl, 3.5 g/L KH_2_PO_4_, 246.5 mg/L MgSO_4_·7H_2_O, 14.7 mg/L CaCl_2_·2H_2_O, 27.8 mg/L FeSO_4_·7H_2_O, and 2 g/L sodium citrate dihydrate. The fed-batch medium contained (per liter) 10 g of glucose, 8 g of (NH_4_)_2_HPO_4_, 13.3 g of KH_2_PO_4_, 1.2 g of MgSO_4_ · 7H_2_O, 1.7 g of citric acid, and 10 mL of a trace metal solution that contained (per liter of 5 M HCl) 10 g of FeSO_4_·7H_2_O, 2.25 g of ZnSO_4_ · 7H_2_O, 1 g of CuSO_4_·5H_2_O, 0.5 g of MnSO_4_·5H_2_O, 0.23 g of Na_2_B_4_O_7_·10H_2_O, 2 g of CaCl_2_ · 2H_2_O, and 0.1 g of (NH_4_)_6_MO_7_O_24_. When necessary, the medium was supplemented with 50 ug/mL kanamycin, 100ug/mL ampicillin, and 17 ug/mL chloramphenicol.

**Table 1:** Strains and plasmids used in the study

Strains and plasmids	Relevant characteristics	Source
*Strains*		
*E. coli* Trans1-T1	F-*φ80(lacZ)ΔM15ΔlacX74hsdR (rk -, mk* +*) ΔrecA1398endA1tonA*	Transgene company
*E. coli* BW25113	*rrnBT14ΔlacZWJ16hsdR514 ΔaraBADAH33 ΔrhaBADL78*	Laboratory storage
*E. coli* BW25113 *∆tnaA*	wild type *∆tnaA*	Laboratory storage
EcSc5HTP	BW25113 *∆tnaA* harboring pBAD-Sa5HTP	This work
EcXc5HTP	BW25113 *∆tnaA* harboring pBAD-Xc5HTP	This work
EcHe5HTP	BW25113 *∆tnaA* harboring pBAD-He5HTP	This work
EcTf5HTP	BW25113 *∆tnaA* harboring pBAD-Tf5HTP	This work
EcCt5HTP	BW25113 *∆tnaA* harboring pBAD-Ct5HTP	This work
EcSaCOMT	BW25113 *∆tnaA* harboring pBAD-SaCOMT	This work
EcOsCOMT	BW25113 *∆tnaA* harboring pBAD-OsCOMT	This work
EcMEL1	BW25113 *∆tnaA* harboring pBAD-MEL1	This work
EcMEL2	BW25113 *∆tnaA* harboring pBAD-MEL2	This work
EcMEL3	BW25113 *∆tnaA* harboring pBAD-Xc5HTP and pZS-MEL1	This work
EcMEL4	BW25113*∆tnaA* harboring pBAD-Xc5HTP and pZS-MEL2	This work
EcHFSaCOMT	BW25113*∆tnaA* harboring pZS-MEL1	
EcHFOsCOMT	BW25113*∆tnaA* harboring pZS-MEL2	
EcMEL5	BW25113*∆tnaA* harboring pBAD-5HTPCOMTsa and pZS-PsmHF	This work
EcMEL6	BW25113*∆tnaA* harboring pBAD-5HTPCOMTos and pZS-PsmHF	This work
EcMEL7	BW25113*∆tnaA* harboring pBAD-XcP4H-OsCOMT and pZS-SaPsmHF-phhBfolM	This work
EcMEL7-1	EcMEL7 + over expression of *metk*	This work
EcMEL7-2	EcMEL7 + over expression of *mtn*	This work
EcMEL7-3	EcMEL7 + over expression of *luxS*	This work
EcMEL7-4	EcMEL7 + over expression of *mtn* and *luxS*	This work
EcMEL7-5	EcMEL7*∆speD*	This work
EcMEL7-6	EcMEL7*∆dcm*	This work
EcMEL8	BW25113*∆tnaA∆speD* harboring pBAD-XcP4H-OsCOMT2 and pZS-SaPsmHF-phhBfolM	This work
EcMELCXPM	BW25113*∆tnaA∆speD* harboring pBAD-OsCOMT2-XcP4H-PhhB-FolM and pZS-SaPsmHF	This work
EcMELCX	BW25113*∆tnaA∆speD* harboring pBAD-OsCOMT2-XcP4H and pZS-SaPsmHF-PhhB-FolM	This work
EcMELCS	BW25113*∆tnaA∆speD* harboring pBAD-OsCOMT2 and pZS-XcP4H -SaPsmHF-PhhB-FolM	This work
EcMELC_T7_S	BW25113*∆tnaA∆speD* (DE3) harboring pBAD-P_T7_OsCOMT2 and pZS-XcP4H -SaPsmHF-PhhB-FolM	This work
EcMELC_tac_S	BW25113*∆tnaA∆speD* harboring pBAD-PtacOsCOMT2 and pZS-XcP4H -SaPsmHF-PhhB-FolM	This work
*Plasmids*		
pKD46	AmpR, λ-Red recombinase expression plasmid, ara-inducible expression, temperature sensitive replication	Laboratory storage
pKD13	Kan^R^, oriR plasmid containing an FRT-aph-FRT cassette	Laboratory storage
pCP20	Amp^R^, Cm^R^, repA(Ts), pSC101 based vector expressing the yeast Flp recombinase	Laboratory storage
pBAD/HisA	Amp^R^, pBR322 origin, araBAD promoter, araC gene	Laboratory storage
pZS	Cm^R^, p15A origin, pBAD based vector expressing recombinase	Laboratory storage
pBAD-Sa5HTP	pBAD containing SaP4H, phhB, FolM genes	This work
pBAD-Xc5HTP	pBAD containing XcP4H, phhB, FolM genes	This work
pBAD-He5HTP	pBAD containing HeP4H, phhB, FolM genes	This work
pBAD-Tf5HTP	pBAD containing TfP4H, phhB, FolM genes	This work
pBAD-Ct5HTP	pBAD containing CtP4H, phhB, FolM genes	This work
pBAD-SaCOMT	pBAD containing SaCOMT	This work
pBAD-OsCOMT	pBAD containing OsCOMT	This work
pBAD-MEL1	pBAD containing SaP4H, phhB, FolM, SaPsmH, SaPsmF, SaCOMT	This work
pBAD-MEL2	pBAD containing SaP4H, phhB, FolM, SaPsmH, SaPsmF, OsCOMT	This work
pZS-MEL1	pZS containing SaPsmH, SaPsmF, SaCOMT	This work
pZS-MEL2	pZS containing SaPsmH, SaPsmF, OsCOMT	This work
pBAD-5HTPCOMTsa	pBAD containing SaP4H, phhB, FolM, SaCOMT	This work
pBAD-5HTPCOMTos	pBAD containing SaP4H, phhB, FolM, OsCOMT	This work
pZS-PsmHF	pZS containing SaPsmH, SaPsmF	This work
pBAD-XcP4H-OsCOMT	pBAD containing SaP4H, OsCOMT	This work
pBAD-XcP4H-OsCOMT2	pBAD containing SaP4H, OsCOMT (C303F, V321T)	This work
pZS-SaPsmHF-phhBfolM	pZS containing SaPsmH, SaPsmF, phhB, FolM	This work
pBADOsCOMT2-XcP4H-PhhB-FolM	pBAD containing OsCOMT2, XcP4H, PhhB, FolM	This work
pBADOsCOMT2-XcP4H	pBAD containing OsCOMT2, XcP4H	This work
pBAD-XcP4H-OsCOMT2	pBAD containing XcP4H, OsCOMT2	This work
pBAD-OsCOMT2	pBAD containing OsCOMT2	This work
pBAD-P_T7_OsCOMT2	pBAD containing P_T7_-OsCOMT2	This work
pBAD-P_tac_OsCOMT2	pBAD containing P_tac_-OsCOMT2	This work

### DNA manipulation

All plasmids used in this study are listed in Table 1 and all primers used in this study for PCR are listed in Additional file 1: Table S1. XcP4H (NCBI Reference Sequence: WP_011035413.1), TfP4H (NCBI Reference Sequence: WP_028838552.1), HeP4H (NCBI Reference Sequence: WP_013332010.1), phhB (GenBank: RMS51283.1), folM (GenBank: EFM2067767.1), SaPsmH (GenBank: AIA00687.1) and SaPsmF (NCBI Reference Sequence: WP_020929557.1) genes were cloned from the corresponding strains. SaP4H (W199F mutant) (NCBI Reference Sequence: WP_016572394.1), CtP4H (W239F mutant) (NCBI Reference Sequence: WP_012354318.1), SaCOMT (NCBI Reference Sequence: WP_016577150.1) and OsCOMT (NCBI Reference Sequence: XP_015650053.1) genes were all codon optimized and synthesized by Generay (Shanghai, China). All plasmids were assembled by Gibson assembly method using the ClonExpress MultiS One Step Cloning Kit (Vazyme, Nanjing China). The construction of plasmids was listed in Additional file 1. The replacement of the native promoter involved in the methionine cycle with the Anderson promoter No. J23108 (ctgacagctagctcagtcctaggtataatgctagc) and the deletion of the *dcm* and *spe*D genes were performed by λ-red recombination. The target sequences were also assembled by the ClonExpress MultiS One Step Cloning Kit. To replace the promoters of the *Mtn, luxS, metF,* and *metK* genes, PCR was performed to obtain the upstream region, the kan resistance gene containing FRT at both ends, the promoter and RBS and the beginning of the gene’s ORF as the downstream region. Then, the PCR products described above were assembled to obtain the target genes. According to the λ-red recombination protocol, various *E. coli* BW25113*∆tnaA* derivatives were constructed. Knockout of *dcm* and *speD* were performed in essentially the same manner as that described above. The target genes used for the deletion of the *dcm* and *speD* genes were made by ligating the upstream region, the kan resistance gene containing FRT at both ends and the downstream region in order.

### Production of melatonin in *E. coli* BW25113*∆tnaA*

The melatonin-producing strains were cultured in 100 mL LB media (500-mL shaking flask) at 37 °C and 200 rpm until the OD_600_ reached 0.6. The enzymes were induced with 0.1% l-arabinose. After incubation at 30 °C and 200 rpm for 8 h, 200 OD cells were harvested by centrifugation at 4 °C and 5000×*g* for 10 min. This induced bacterial pellet was suspended into 20 mL M9Y media (50-mL flask) containing 2 g/L tryptophan, 4 g/L methionine (and 3% glycerin when necessary) and grown at 30 °C and 200 rpm. Samples were collected at 3 h, 6 h, 12 h, 24 h, 48 h, 72 h, and 96 h, and the concentrations of 5-HTP, NAS and melatonin were analyzed by HPLC. Acetyl Coenzyme A kit (Solarbio, Beijing, China) was used to detect the concentration of acetyl coenzyme A. Bioscreen C (Lab Systems Helsinki, Finland) was used to measure the grow curve of *E. coli* BW25113.

### Production of 5-HTP in *E. coli* BW25113*∆tnaA*

*E. coli* BW25113 *∆tnaA* was transformed with the pBAD-Sa5HTP, pBAD-Xc5HTP, pBAD-Tf5HTP, pBAD-He5HTP and pBAD-Ct5HTP plasmids. The culture methods were the same as described above (production of melatonin), and 200 OD-induced cultures were harvested and suspended into 20 mL of M9Y medium containing 2 g/L tryptophan and grown at 30 °C and 200 rpm. Samples were collected at the time points described above and analyzed by HPLC.

### Fed-batch fermentation and optimization

A single colony of Ec-MEL8 from a cell plate was inoculated into 5 mL LB media and cultured at 37 °C and 200 rpm overnight. Then, 1% (v/v) of the culture was transferred into 100 ml LB media (500-mL flask) and grown at 37 °C and 200 rpm for approximately 8–10 h. 5% (v/v) of the cultures were transferred to a 2-L bioreactor (BXBIO, Shanghai, China) with 1 L fed-batch media. The pH was controlled at 6.8 by automatic feeding of 30% (v/v) NH_4_OH, and the temperature was set at 37 ℃. The dissolved oxygen concentration was maintained above 20% air saturation by supplying air at 1 vvm (air volume/working volume/minute) and by automatically controlling the agitation speed up to 700 rpm. When the initial 10 g/L glucose was consumed, a feeding solution containing 500 g of glucose and 10 g of MgSO_4_ · 7H_2_O per liter was periodically added. When the OD_600_ reached 20, the culture was induced by adding L-arabinose to a final concentration of 1 g/L, the temperature was set at 30℃, and a solution of 4 g/L tryptophan, 6 g/L methionine and 3% (v/v) glycerol was added to the media. Samples were collected to measure the biomass concentrations (OD_600_) and glucose concentrations (SBA-40E biosensor analyzer (Institute of Biology, Shandong Province Academy of Sciences, China)) and for HPLC analysis. For optimized conditions, the culture was induced when OD_600_ reached 40 and after 12 h induction, a solution of 6 g/L tryptophan, 10 g/L methionine and 3% (v/v) glycerol was added to the media.

### HPLC analysis and LC/MS

l-tryptophan from Sigma (St. Louis., USA), 5-HTP from Aladdin (Shanghai, China), NAS from Sigma and melatonin from Aladdin (Shanghai, China) were used as the standards. The standards and the supernatant concentration of tryptophan, 5-HTP and NAS were measured after dilution of the samples with methanol/water (15:85 v/v). For analysis of melatonin, standard and supernatant of cultures were diluted to a final concentration of methanol/water (40:60 v/v). The samples were filtered through a 0.22-μm nylon filter and then analyzed by HPLC (Agilent 1260 series, Hewlett-Packard) using an Agilent ZORBAX Eclipse Plus C18 column (4.6 × 100 mm, 3.5-Micron). Tryptophan, 5-HTP and NAS were quantified under 275 nm UV detection with methanol/water (15:85 v/v) as the mobile phase. Melatonin was quantified under 290 nm UV detection with methanol/water (40:60 v/v) as the mobile phase. NAS and melatonin were identified by QTRAP 6500 and AB SCIEX using Gemini 3 µm NX-C18 110 Å (50 × 2 mm) with 95% methanol (containing 0.1% formic acid) and 5% water (containing 0.1% formic acid) (v/v).

## Results and discussion

### Conceptual design of the melatonin biosynthetic pathway by virtue of the physostigmine pathway

**Fig. 1:**
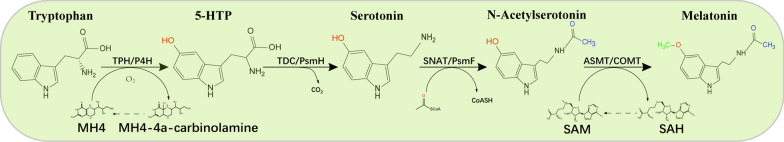
The melatonin biosynthesis pathway. TPH, tryptophan hydroxylase; 5-HTP, 5-Hydroxytryptophan; TDC, tryptophan decarboxylase; SNAT, serotonin *N*-acetyltransferase; ASMT, *N*-acetylserotonin methyltransferase; COMT, caffeic acid *O*-methyltransferase; SAM, S-adenosylmethionine; SAH, S-adenosylhomocysteine

When searching tryptophan derivatives in prokaryotes, we found three intermediates of physostigmine biosynthesis (5-HTP, 5-HT, and NAS) in *Streptomyces albulus* are shared by melatonin biosynthesis. Enlightened by the physostigmine biosynthetic pathway, a novel biosynthetic pathway for melatonin production was designed (Fig. 1). In the first catalytic step of physostigmine biosynthesis, the production of 5-HTP was proposed to be catalyzed by TPH. However, the gene encoding TPH was not included in the physostigmine biosynthesis gene cluster and was not specifically identified in the report. In animals, TPH and phenylalanine 4-hydroxylase (P4H) are two subgroups of aromatic amino acid hydroxylases (AAAHs) that share high sequence similarity. BLASTP analysis of the TPH homolog in the *S. albulus* genome revealed a putative AAAH, which has typical characteristics of P4H (SaP4H). It was reported that some bacterial P4Hs exhibit activity towards both phenylalanine and tryptophan. Moreover, the substitution of a small number of residues (e.g., W179F substitution) causes the preferred substrate of P4H to change from phenylalanine to tryptophan [22]. Therefore, SaP4H was used to establish the 5-HTP biosynthetic pathway. Alternative P4Hs from other bacteria, including *Xanthomonas campestris* [22], *Halomonas elongata*, *Thermomonas fusca*, and *Cupriavidus taiwanensis* [27]*,* were also selected to catalyze the first step of melatonin biosynthesis. The genes encoding homologs of PsmH and PsmF were selected from *S. albulus* to establish the second and third catalytic steps of melatonin biosynthesis. For the final step of melatonin biosynthesis, two kinds of enzymes: ASMT and COMT have been identified to exhibit the catalytic activity. ASMT, as a methyltransferase initially identified in animals and plants, had low enzyme activities, indicating its role as a rate-limiting enzyme in melatonin synthesis [28]. Besides, ASMT was noncompetitively inhibited by NAS and melatonin and competitively inhibited by its product S-adenosylhomocysteine (SAH) [29, 30]. Compared with ASMT, COMT, which is a methyltransferase found in plants [31], also O-methylates NAS to melatonin with a much higher catalytic capacity (100 times) than that of ASMT [20, 32]. Therefore, COMT from *Oryza sativa* (OsCOMT) was the preferred enzyme, and this enzyme exhibits the highest activity for converting NAS to melatonin as ever reported [12]. In *S. albulus*, BLASTP analysis revealed a putative gene encoding COMT, which shared the highest sequence identity with OsCOMT. The resulting SaCOMT and OsCOMT were then selected to establish the final catalytic step of melatonin biosynthesis.

Above all, we designed a new melatonin biosynthetic pathway in *E. coli* based on the physostigmine biosynthetic pathway. All four proteins from *S. albulus*, namely, SaP4H, SaCOMT, SaPsmF and SaPsmH, were expressed well as soluble recombinant proteins in *E. coli*. Among these genes, the genes encoding SaP4H and SaCOMT were optimized according to the *E. coli* codon preference, while the genes encoding SaPsmF and SaPsmH were not optimized. The 5-HTP biosynthetic module and COMT were involved in the first and final steps of melatonin biosynthesis. According to previous reports, these two steps were proposed to be the rate-limiting of melatonin production and were tested first [9, 20].

### Engineering the biosynthetic module to produce 5HTP in *E. coli*

**Fig. 2:**
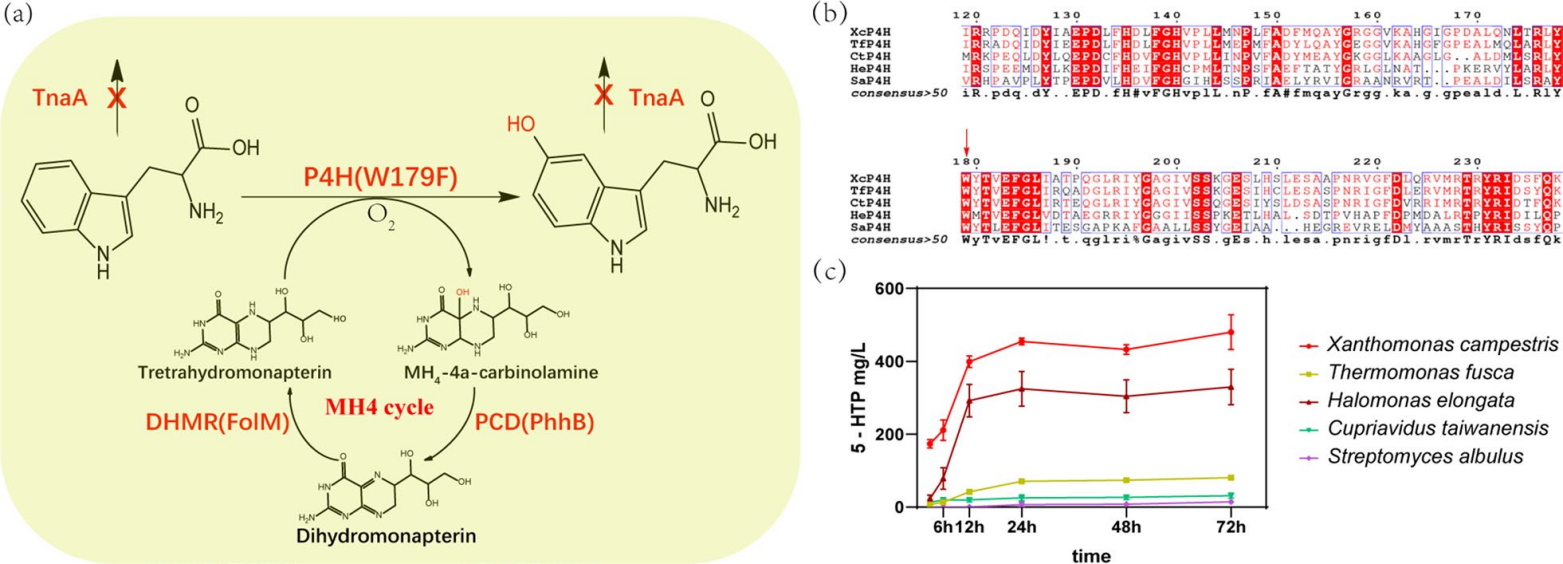
Prokaryotic P4Hs catalyzed the formation of 5-HTP in *E. coli*. **a** The biosynthetic pathway of 5-HTP constructed in *E. coliΔtnaA*. **b** Partial results of P4H alignment. The sequences and functional domains of P4Hs were highly conserved, and the arrows indicate the site requiring the mutation of tryptophan to phenylalanine. **c** Effect of P4H on 5-HTP production in recombinant *Escherichia coli*. 5-HTP levels were subjected to HPLC, and the data are the means ± standard deviations of triplicate experiments

The 5-HTP biosynthetic module was engineered in three ways (Fig. 2a). First, the W179F substitution was discovered to change the preferred substrate of P4H from phenylalanine to tryptophan and hence to enhance its TPH activity. Thus, the W179F substitution was introduced into various P4Hs (Fig. 2b), and the resulting genes encoding the W179F mutant were cloned into the pBAD vector under the control of the arabinose-inducible araBAD promoter. Second, a tetrahydromonapterin (MH4) recycling system was constructed to provide cofactor to P4Hs. The genes encoding PhhB from *P. aeruginosa* and FolM from *E. coli* were cloned and inserted downstream of the genes encoding the W179F mutants of P4Hs in the pBAD plasmid (Fig. 2a). The resulting plasmids were named pBAD-Sa5HTP, pBAD-Xc5HTP, pBAD-Tf5HTP, pBAD-He5HTP and pBAD-Ct5HTP. Third, the *E. coli* mutant strain (abbreviated Ec*∆tnaA*), in which the *tnaA* gene encoding tryptophanase was deleted to block the degradation of tryptophan and 5-HTP, was generated (Fig. 2a). The pBAD-5HTP plasmids were transformed into the Ec*∆tnaA* strain*,* and all the recombinant proteins were well expressed (Additional file 1: Fig. S1a). The whole-cell biocatalysis of tryptophan to 5-HTP in these strains at different intervals was analyzed and compared. As shown in Fig. 2c, all the strains could produce 5-HTP after the addition of tryptophan, confirming the successful establishment of the 5-HTP biosynthesis module. However, the strain harboring the putative SaP4H from *S. albulus* displayed the lowest level of 5-HTP production. It seemed that the putative P4H-W199F of *S. albulus* might exhibit lower catalytic activity toward tryptophan. It is also possible that other unknown tryptophan hydroxylases were involved in the conversion of tryptophan to 5-HTP during physostigmine biosynthesis. The XcP4H-W179F exhibited superior catalytic activity toward tryptophan (Fig. 2c), which is in accordance with a previous report. It produced ⁓500 mg/L 5-HTP in the shake flask with tryptophan supplied. Thus, XcP4H-W179F was used as the 5-HTP biosynthetic module in subsequent pathway engineering.

### COMT overexpression enabled melatonin production from NAS

**Fig. 3:**
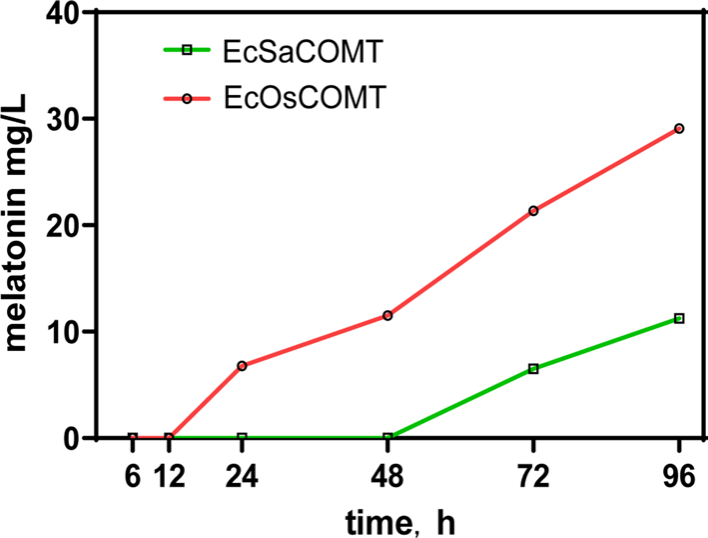
Comparison of the effects of OsCOMT and SaCOMT on melatonin production. Two *E. coli* strains (10 OD) were harvested after 16 h of induction at 16 °C, resuspended in 1 mL pH 7.0 Tris–HCl, and cultured with 1 mg/mL NAS and 2 mg/mL methionine at 30 °C. Data are the means ± standard deviations of triplicate experiments. The absence of error bars indicates that the error was smaller than the symbol size

Two genes encoding COMT from *S. albulus* and *O. sativa* were singly cloned into the pBAD vector under the control of the araBAD promoter. The resulting pBAD-SaCOMT and pBAD-OsCOMT plasmids were transformed into the strain Ec*∆tnaA*, resulting in EcSaCOMT and EcOsCOMT, respectively, and the soluble proteins were obtained (Additional file 1: Fig. S1b). By adding NAS and methionine, the capabilities of the two COMTs in the biocatalysis of NAS to melatonin were tested. As shown in Fig. 3, both COMTs facilitated the conversion of NAS to melatonin, suggesting that both COMTs could be used in engineering a pathway for melatonin production. The strain harboring OsCOMT produced significantly higher levels of melatonin than the strain harboring SaCOMT, and SDS-PAGE analysis showed that the expression of OsCOMT was much higher than that of SaCOMT. Two COMT genes were coupled with other intermediate product synthesis genes to be constructed on the co-expression plasmid to verify their ability to increase the production of melatonin. As far as we know, this was the first reported COMT protein identified in microorganisms, which has expanded the COMT candidate gene pool involved in melatonin biosynthesis. Combined with SaP4H, SaPsmH and SaPsmF, a putative pathway of melatonin synthesis in *S. albulus* was identified.

### Melatonin production pathway engineering

**Fig. 4:**
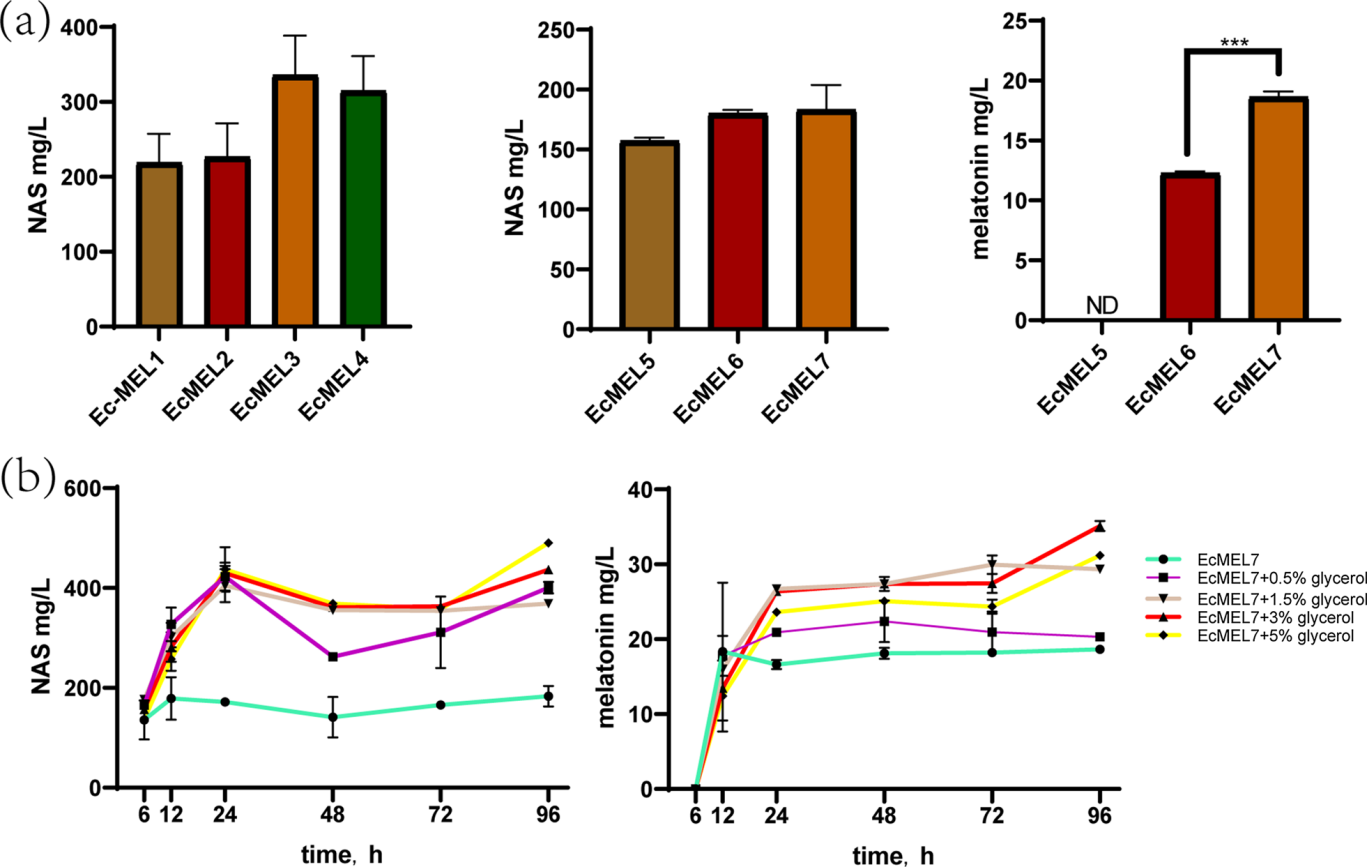
EcMEL1-7 strains catalyzed the production of NAS and melatonin and the effect of different concentrations of glycerol in conversion medium. **a** Production of NAS and melatonin by fermentation of the EcMEL1-7 strains for 96 h in shake flasks as described in method 2. **b** The production of NAS and melatonin by fermentation of EcMEL7 with different concentrations of glycerol. Data are the means ± standard deviations of triplicate experiments. The absence of error bars indicates that the error was smaller than the symbol size

Based on the 5-HTP biosynthetic module and the COMT from *S. albulus* or *O. sativa*, a complete melatonin production pathway was constructed (Additional file 1: Fig. S2). With all the genes responsible for melatonin biosynthesis expressed from a single pBAD plasmid in the Ec*∆tnaA* strain, the recombinant strains EcMEL1 and EcMEL2 (with either SaCOMT or OsCOMT) were assembled. Besides, another two strains, namely EcMEL3 and EcMEL4, were made by placing the genes encoding SaPsmH, SaPsmF and COMT on another plasmid (pZS), with the 5-HTP biosynthetic module on the pBAD plasmid. Unexpectedly, melatonin was not produced in all these four strains, while significant NAS accumulation was observed (Fig. 4a). Results of SDS-PAGE (Additional file 1: Fig. S3) showed that the proteins necessary for the synthesis of melatonin were well expressed, except for the last enzyme, COMT, which catalyzed NAS to melatonin. These results indicated that the weak or absent expression of COMT might restricted the production of melatonin. To improve the expression of COMT in the recombinant strains, a series of plasmid modifications were carried out (Additional file 1: Fig S2C–G). When we moved COMT from pZS plasmid to pBAD plasmid, a clear OsCOMT band was observed and a total of 12.29 ± 0.13 mg/L melatonin was produced by Ec-MEL6 in flasks, while no obvious melatonin or SaCOMT was produced by EcMEL5 (Fig. 4a, Additional file 1: Fig S4a). Thus, OsCOMT is more superior for the bioconversion of NAS to melatonin than SaCOMT in this system. Whereas the synthesis of melatonin was still blocked at the step catalyzed by COMT. During the whole-cell biocatalytic, the production of NAS in EcMEL6 was nearly 200 mg/L, which was about 15-fold of melatonin. This indicated that COMT expression is of vital importance for melatonin production.

In order to enhance the expression of OsCOMT and achieved higher melatonin yields, the next melatonin-producing strain, EcMEL7, was made by placing the gene encoding OsCOMT immediately downstream of the XcP4H. Results showed that the production of melatonin by the EcMEL7 strain was increased to 18.64 ± 0.46 mg/L (Fig. 4a). SDS-PAGE analysis confirmed that the expression of OsCOMT was enhanced in the EcMEL7 cells (Additional file 1: Fig S4b). To further increase the expression of OsCOMT, we also shifted OsCOMT to the front of XP4H or put OsCOMT singly on the pBAD plasmid (Additional file 1: Fig S2J). However, when OsCOMT was shifted to the upstream of XP4H, the recombinant strains did not show an increased in COMT expression but had a significantly decreased soluble expression of XP4H (Additional file 1: Figure S4c), which caused the synthesis of melatonin blocked in the first step. When putting OsCOMT on the pBAD plasmid alone, and even replacing Para with a stronger promoter such as P_T7_ or P_tac_, the expression of COMT and the production of melatonin were not increased compared to that of EcMEL7 (Additional file 1: Figure S5a, b). It seemed that the expression of COMT has reached its limit in the current six enzymes co-expression system. Even when the gene elements involved in the COMT expression were enhanced, a stronger expression would not be achieved due to the limitation of resources related to transcription and translation.

### Improved production of melatonin by Cofactor engineering and protein engineering

After the establishment of the complete melatonin biosynthetic pathway, cofactor engineering and protein engineering were conducted to further enhance melatonin production. First, cofactor engineering was conducted to increase the supply of acetyl-CoA and SAM, resulting in a significant increase in melatonin production. Acetyl–CoA is an important cofactor of PsmF that is involved in the biosynthesis of NAS from 5-HT. HPLC analysis of the supernatant of EcMEL7 showed a large amount of 5-HT accumulation (data not show). The low conversion efficiency of 5-HT to NAS might indicate the lack of acetyl-CoA. Glucose, acetate and glycerol were reported to be able to provide energy and the acetyl group for acetyltransferase in *E. coli* [33, 34]. 10 g/L glucose, 50 mM acetate or 5% glycerol was added to M9 medium and the whole-cell biocatalytic efficiency of EcMEL7 was evaluated. Results showed that glycerol effectively increased the yield of NAS compared with that of glucose and acetate (Additional file 1: Fig. S6). As shown in Additional file 1: Fig S7, the level of Acetyl Coenzyme A in the supernatant was increased by 55% after adding glycerol, which confirmed that glycerol augmented the PsmF activity by facilitating the availability of Acetyl Coenzyme A cofactor. Therefore, different concentrations of glycerol were tested in the whole-cell bioconversion of melatonin from tryptophan. The highest production of NAS was 569.82 ± 16.88 mg/L in the shake flask (Fig. 4b). Moreover, the addition of glycerol also significantly increased the production of melatonin (Fig. 4b). The increase in the production of melatonin was glycerol concentration-dependent. The highest level of melatonin was achieved: 35.13 ± 0.66 mg/L (Fig. 4b) when 3% glycerol was added into the whole-cell catalysis system. Thus, subsequent optimization experiments all added 3% glycerol.

**Fig. 5:**
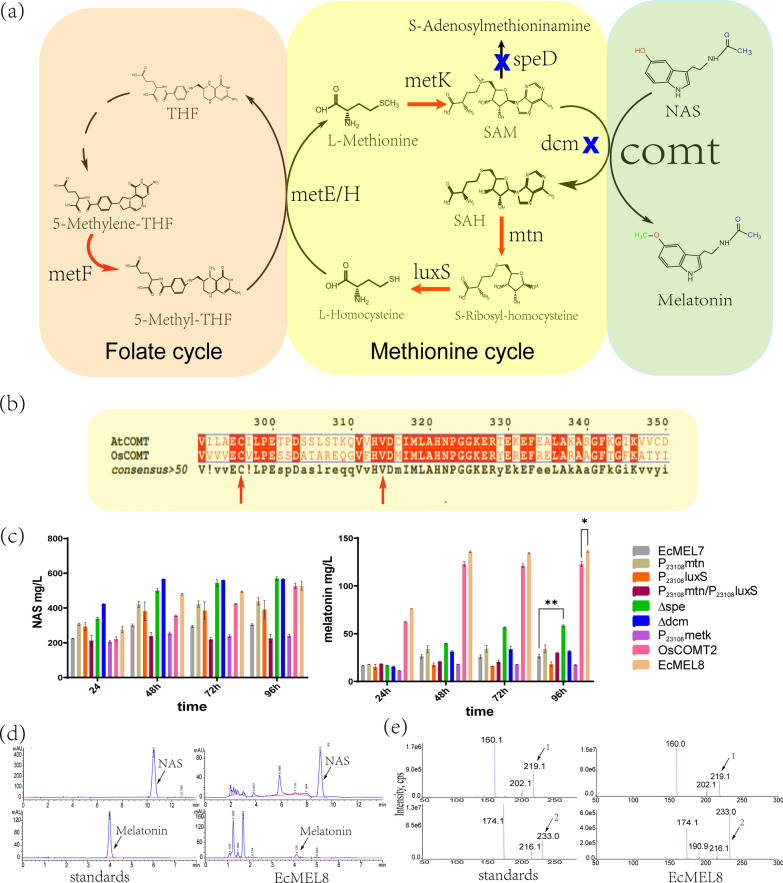
Cofactor engineering and protein engineering for high-level production of melatonin. **a** Metabolic engineering for cofactor SAM of COMT. The red arrow indicates the enhanced expression of the gene, and the blue cross indicates the knockout of the gene. **b** Alignment of the OsCOMT and AtCOMT protein sequences. **c** Conversion of tryptophan to NAS and melatonin by recombinant *E. coli* strains. **d** HPLC analysis of the standard and bioconversion products of the representative EcMEL8 strain. **e** LC–ESI–MS analysis of symbols from panel d: exact mass of compound 1[M + H]^+^[*m/z*](219.1), compound 2[M + H]^+^[*m/z*](233.0). Data are the means ± standard deviations of triplicate experiments

Radical *S*-adenosylmethionine (SAM), the other important cofactor, plays a key role in the process of COMT catalysis. SAM is a common methyl donor in vivo that participates in the rate-limiting steps of the biosynthesis of multiple compounds and undergoes many genome modifications to remove inhibition [35]. Research on the enzyme activity of COMT in plants showed that it was positively correlated with the SAM/SAH ratio [35–37]. To test whether increased SAM levels and decreased SAH accumulation could improve the potential of COMT and hence enhance the production of melatonin in *E. coli*, a series of genes related to SAM recycling were deleted or overexpressed (Fig. 5a). Among the deleted and overexpressed genes, deletion of the *speD* gene increased the production of melatonin by two-fold (Fig. 5c). The SpeD catalyzed decarboxylation of SAM to produce dSAM: one of the precursors involving spermidine synthesis. Deletion of *speD* gene reduced the consumption of SAM and provided more methyl donors to enhance melatonin synthesis. In addition, *speD* knockout has no significant effect on the growth of BW25113Δ*tnaA* (Additional file 1: Fig S8). The modification of *mtn*, *luxS*, and *dcm* increased the production of NAS, but does not caused significant differences (p > 0.05) in melatonin production (Fig. 5c).

Wang et al*.* reported that amino acid substitutions near the NAS-binding pocket (C296F, Q310L, and V314T) significantly enhanced the catalytic activity of COMT from *Arabidopsis thaliana* (AtCOMT) [38]. The corresponding residues of C296 and V314 are conserved in OsCOMT, while Q310 is not (Fig. 5b). Thus, the corresponding substitutions of C296F and V314T were introduced to OsCOMT, yielding OsCOMT2. OsCOMT2 increased melatonin production to 122.83 ± 4.44 mg/L, which was fivefold higher than the melatonin production with OsCOMT (Fig. 5c). By combining the mutation of OsCOMT and the deletion of *speD*, the EcMEL8 strain was obtained. EcMEL8 produced 136.17 ± 1.33 mg/L melatonin and 879 ± 71.42 mg/L NAS in a shake flask (Fig. 5c). Figure 5d showed the HPLC analysis of the EcMEL8 fermentation supernatant, and the target products were confirmed by LC–MS (Fig. 5e). The results showed that almost all the consumed tryptophan (4.94 mM) was converted into NAS and melatonin (4.12 mM + 0.56 mM), and other tryptophan derivatives were negligible. As reported by Luo et al., the engineered strain produced high levels of byproduct AcTRPM when achieved high yields of melatonin in the fed-batch fermentation process with tryptophan as substrate, due to the TRPM-forming activity of the TDC (tryptophan decarboxylase) [21]. However, the PsmH and PsmF proteins from the physostigmine pathway were very specific to their substrates [23], and the EcMEL8 strain did not produce obvious byproducts. Compared to the EcMEL7 strain, the yield of melatonin produced by EcMEL8 was effectively improved and the ratio of NAS/melatonin production was reduced, suggested that the bioconversion of NAS to melatonin was successfully enhanced. Nevertheless, the fivefold higher level of NAS than melatonin in EcMET8 indicated that COMT was still the rate-limiting step for the production of melatonin in this study.

### High-level melatonin production in a fermenter

**Fig. 6:**
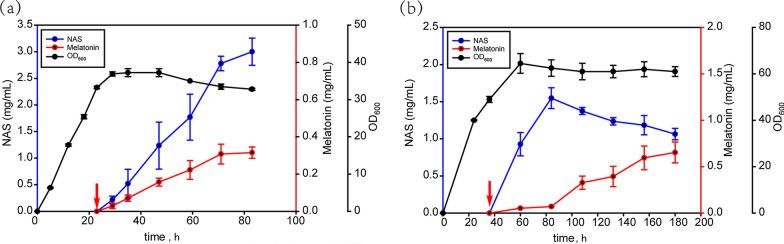
High-level melatonin production in a fermenter. **a** Fed-batch fermentation of EcMEL8. The cultures were added 0.1% L-arabinose at 18 h. The red arrow indicates the time (23 h) at which tryptophan, methionine, and 3% glycerol were added to the fermenter. The NAS and melatonin concentrations were measured at 29 h, 35 h, 47 h, 59 h, 71 h, and 83 h. **b** Fed-batch fermentation of EcMEL8 after optimization. Those were induced at 24 h with 0.1% l-arabinose. Tryptophan, methionine, and 3% glycerol were added to the fermenter at 36 h. The NAS and melatonin concentrations were measured at 60 h, 84 h, 108 h, 132 h, 156 h, and 180 h. (n = 2)

Whole-cell bioconversion of tryptophan to melatonin in EcMET8 was also performed in a 2-L fermenter with a higher cell density (OD_600_ = 38). Melatonin yield reached its highest level in the medium of 0.32 ± 0.03 g/L at 71 h with the synthesis efficiency of melatonin produced via per g of dry cell weight (DCW) at 35 mg/g DCW. NAS production continued to increase even after 83 h and reached 3.0 ± 0.26 g/L (Fig. 6a). Thus, both melatonin and NAS production were significantly higher in this system than in shake flasks. The cell transformation efficiency was reduced compared with that of the shake flask. This may be due to the limitations of the fermentation process. After the optimization of fermentation conditions, Fig. 6b showed that the accumulation of NAS decreased to 1.06 ± 0.07 g/L, and the melatonin production finally reached 0.65 ± 0.11 g/L at 180 h. The higher OD value, as expected, brought a higher production of melatonin. Under the optimized fermentation conditions, the melatonin synthesis efficiency of EcMEL8 reached 59 mg/g DCW, which was even higher than the strain reported by Luo et al. However, the limitation of the final step of melatonin synthesis in EcMEL8 was not completely eliminated: high levels of NAS accumulated in the fermenter. Subsequent further work should focus on removing the problem of NAS accumulation to improve melatonin production.

## Conclusions

In this study, the melatonin synthesis pathway in *Streptomyces albicans* was identified, and a new melatonin biosynthesis pathway in *Escherichia coli* was successfully constructed on this basis. By balancing heterologous proteins expression elements, optimizing cofactors supplementation, and modifying the rate-limiting enzyme COMT, the production of melatonin was increased by 11-fold. In this study, the gene resource library of the melatonin synthesis pathway was extended to prokaryotic genes, which provided a fast and excellent synthesis pathway for the production of tryptophan derivatives in genetically engineered strains.

## Supplementary Information

**Additional file 1.** Supplementary Fig. 1. (a), SDS-PAGE analysis of pBAD-5HTPs. The red arrow indicates the expression of P4Hs. (b), SDS-PAGE analysis of EcSaCOMT and EcOsCOMT. Supplementary Fig. 2. Construction of plasmids for expression of melatonin-related proteins. Supplementary Fig 3. SDS-PAGE analysis of EcMEL1, EcMEL2, EcMEL3, and EcMEL4. Supplementary Fig 4. SDS-PAGE analysis of EcMEL5, EcMEL6, EcMEL7, EcMELCX and EcMELCXPM. Supplementary Fig. 5. Comparison of the melatonin production by EcMEL7, EcMELCS, EcMEL-CtacS and EcMEL-CT7S. Supplementary Fig 6. Comparison of adding glucose, acetate and glycerol in M9Y medium for biosynthesis of NAS and melatonin. Supplementary Fig. 7. The concentration of Acetyl Coenzyme A in EcMEL7 and EcMEL7+5% glycerol during the whole-cell biocatalysis. Supplementary Fig. 8. The grow curve of BW25113*ΔtnaA* and BW25113*ΔtnaA ΔspeD*. Table S1. Primers used in DNA manipulation. Table S2. The OD_600_ of Melatonin Producing Strains in M9Y medium. Table S3. The OD_600_ of Melatonin Producing Strains after adding glucose, glycerol, and acetate.
