# Supplementary material for: Combining protein and metabolic engineering strategies for biosynthesis of melatonin in Escherichia coli
Source: Microb Cell Fact. 2021 Aug 28;20:170. doi: 10.1186/s12934-021-01662-8 (PMC8403405; doi:10.1186/s12934-021-01662-8)
Supplement: Supplementary file 1 — Additional file 1. Supplementary Fig. 1. (a), SDS-PAGE analysis of pBAD-5HTPs. The red arrow indicates the expression of P4Hs. (b), SDS-PAGE analysis of EcSaCOMT and EcOsCOMT. Supplementary Fig. 2. Construction of plasmids for expression of melatonin-related proteins. Supplementary Fig 3. SDS-PAGE analysis of EcMEL1, EcMEL2, EcMEL3, and EcMEL4. Supplementary Fig 4. SDS-PAGE analysis of EcMEL5, EcMEL6, EcMEL7, EcMELCX and EcMELCXPM. Supplementary Fig. 5. Comparison of the melatonin production by EcMEL7, EcMELCS, EcMEL-CtacS and EcMEL-CT7S. Supplementary Fig 6. Comparison of adding glucose, acetate and glycerol in M9Y medium for biosynthesis of NAS and melatonin. Supplementary Fig. 7. The concentration of Acetyl Coenzyme A in EcMEL7 and EcMEL7+5% glycerol during the whole-cell biocatalysis. Supplementary Fig. 8. The grow curve of BW25113ΔtnaA and BW25113ΔtnaA ΔspeD. Table S1. Primers used in DNA manipulation. Table S2. The OD600 of Melatonin Producing Strains in M9Y medium. Table S3. The OD600 of Melatonin Producing Strains after adding glucose, glycerol, and acetate. [file 12934_2021_1662_MOESM1_ESM.docx]

Additional file

**Combining Protein and Metabolic Engineering Strategies for biosynthesis of melatonin in *Escherichia coli***

Yanfeng Zhang ^1,2†^, Yongzhi He ^1†^, Nan Zhang ^1,2^, JiaJia Gan ^1,2^, Shan Zhang ^3*^, and Zhiyang Dong ^1,2*^

*1. State Key Laboratory of Microbial Resources, Institute of Microbiology, Chinese Academy of Sciences, Beijing, 100101, People’s Republic of China*

*2. University of Chinese Academy of Sciences, Beijing, 100049, People’s Republic of China*

*3. SHENZHEN SIYOMICRO BIO-Tech CO., LTD, Shenzhen, 518116, People’s Republic of China*

*. Corresponding author: Dr. Zhiyang Dong: dongzy@im.ac.cn, Address: NO. 1 West Beichen Road, Chaoyang District 100101, Beijing, China

*. Corresponding author: Dr. Shan Zhang: zhangs@outlook.com, Address: SHENZHEN SIYOMICRO BIO-Tech CO., LTD, NO. 39 Qingfeng Avenue, Baolong Community, Longgang District Shenzhen 518116, People’s Republic of China.

^†^. These authors contribute equally to this work.

**Construction of plasmids**

Plasmid DNA assemblies were performed by Gibson Assembly. For the construction of the pBAD-Sa5HTP plasmid, the SaP4H gene was amplified by PCR with the oligonucleotide primers SaP4H-F and SaP4H-R using pET21-SaP4H as the template. The *phhB* gene from *P. aeruginosa* and the *folM* gene from *E. coli* were cloned by PCR with the oligonucleotide primers phhB-F/phhB-R and FolM-F/FolM-R through the homologous recombination method and subsequently inserted downstream of P4Hs in the pBAD/HisA plasmid. The resulting plasmid, pBAD-Sa5HTP, contained the SaP4H, PhhB, and FolM genes under the control of the araBAD promoter. The XcP4H, TfP4H, HeP4H and CtP4H genes were amplified with the primers XcP4H-F/XcP4H-R, TfP4H-F/TfP4H-R, HeP4H-F/HeP4H-R, and CtP4H-F/CtP4H-R using the corresponding genomes or synthetic DNA. The 5.1-kb plasmid fragment was amplified from pBAD-Sa5HTP with the primers phhB-F/pBAD-R. Then, phenylalanine hydroxylase from different sources was inserted into a 5.1-kb vector, and the resulting plasmids were named pBAD-Xc5HTP, pBAD-Tf5HTP, pBAD-He5HTP and pBAD-Ct5HTP. To amplify the SaCOMT and OsCOMT genes, PCR was performed with the primers pBAD-SaCOMT-F/pBAD-SaCOMT-R and pBAD-OsCOMT-F/pBAD-OsCOMT-R using the pET-21-SaCOMT and pET-21-OsCOMT plasmids as templates, and the genes were ligated into pBAD/HisA. For the construction of pBAD-MEL1, the SaPsmH, and SaPsmF genes were amplified with the primers SaPsmH-F/SaPsmH-R and SaPsmF-F/SaPsmF-R using the genomic DNA of *Streptomyces albulus*. SaCOMT gene was amplified with the primers SaCOMT-F/pBAD-SaCOMT-R. The plasmid backbone of pBAD-MEL1 was amplified with the primers FolM-R/pBAD-F using the pBAD-Xc5HTP plasmid as the template. The resulting pBAD-MEL1 plasmids containing XcP4H, PhhB, FolM, SaPsmH, SaPsmF and SaCOMT all had their own RBS successively under the control of the araBAD promoter. To generate pBAD-MEL2, the OsCOMT gene was amplified with the primers OsCOMT-F/pBAD-OsCOMT-R instead of SaCOMT, and the gene was ligated with an 8.1-kb fragment of pBAD-MEL1.

The pZS plasmid was modifid from pBAD/HisA: ori and resistance genes of pBAD were changed to coexist with the pBAD plasmid. To amplify the SaPsmHF genes, PCR was performed with the primers SaPsmH-F and SaPsmF-R using the plasmid pBAD-MEL1 as the template. The COMT genes were amplified with the primers pZSSaCOMT-F/pZSSaCOMT-R and pZSOsCOMT-F/pZSOsCOMT-R using each template, and a the 4-kb fragment was amplified from the pZS plasmid with the primers pZS-F/pZS-R. Then, two sequences were assembled with a 4-kb fragment, resulting in pZS-MEL1 and pZS-MEL2. To generate pBAD-5HTPCOMTsa and pBAD-5HTPCOMTos, PCR was performed with the primers FolM-R2/pBAD-F using pBAD-5HTP as the template to obtain a 7.0-kb fragment, and this fragment was ligated with COMTs, which was amplified with the primers OsCOMT-F2/pBAD-OsCOMT-R and SaCOMT-F2/pBAD-SaCOMT-R using a preserved plasmid as the template. The pZS-PsmHF plasmid was generated by self-ligating the PCR product, which was amplified with the primers PsmF-R/pZH-F2 using pZS-MEL3 as the template. To generate pBAD-XcP4H-OsCOMT, the OsCOMT gene was amplified with the primers OsCOMT-F3/pBAD-OsCOMT-R and ligated into the 4.8-kb fragment, that pBAD plasmid contained XcP4H, which was amplified with the primers XcP4H-R/pBAD-F using pBAD-Xc5HTP as the template. For the amplification of the PhhB and FolM genes, PCR was performed with the primers PhhB-F/FolM-R, and the products were ligated with a 6.1-kb fragment of pZS-PsmHF to generate the pZS-SaPsmHF-phhBfolM plasmid. PCR was performed with the primers OsCOMTmutant-F/OsCOMTmutant-R using pBAD-xcP4H-OsCOMT as the template, and the plasmid was transformed into *E. coli* trans1-T1 to obtain pBAD-XcP4H-OsCOMT (C303F). Then, to generate pBAD-XcP4H-OsCOMT2, PCR was performed with the OsCOMTmutant-F2/OsCOMTmutant-R2 primers using pBAD-xcP4H-OsCOMT(C303F) as the template, and the plasmid was transformed into *E. coli* trans1-T1.

Table S1. Primers used in DNA manipulation

| **Name** | **Sequences (5’ to 3’)** |
| --- | --- |
| SaP4H-F | GCTAACAGGAGGAATTAACCATGTCGACCATGCGTAAACGTAC |
| SaP4H-R | ATGTATATCTCCTTCTTAAAGTTAAATTAACCACGATCACGCGGCAG |
| phhB-F | TTTAAGAAGGAGATATACATGACCGCACTCACCCAAGC |
| phhB-R | GTATATCTCCTTCTTAAAGTTAAATTATTTGCGCCCCTCGGCGG |
| FolM-F | TTAACTTTAAGAAGGAGATATACATGGGTAAAACCCAGCCCTT |
| FolM-R | CCGCCAAAACAGCCAAGCTTTTAACGCAGATGACGACCGC |
| PBAD-F | AAGCTTGGCTGTTTTGGCGG |
| PBD-R | GGTTAATTCCTCCTGTTAGCCCAAAAA |
| XcP4H-F | GCTAACAGGAGGAATTAACCATGAACACAGCGCCGCGCCGC |
| XcP4H-R | TATATCTCCTTCTTAAAGTTAAATTACACGTCGCCGTCGCGGCTC |
| TfP4H-F | CTAACAGGAGGAATTAACCATGAGCACCGCCGCGCCGCGT |
| TfP4H-R | TATATCTCCTTCTTAAAGTTAAATTAGATATCACCGTCCGCAGCCCAAC |
| HeP4H-F | CTAACAGGAGGAATTAACCATGAAGGCACATGACGAC |
| HeP4H-R | GTATATCTCCTTCTTAAAGTTAAATTAGGCACTGGCGGCTTC |
| CtP4H-F | GCTAACAGGAGGAATTAACCATGCGCATTATCTGTATGATCTACCGT |
| CtP4H-R | ATGTATATCTCCTTCTTAAAGTTAAATTAGATATCCTCCGTGTCCGCCCAA |
| pBAD-SaCOMT-F | GGCTAACAGGAGGAATTAACCATGCCAGACGACCCGAGC |
| pBAD-SaCOMT-R | CCGCCAAAACAGCCAAGCTTTTAGACCGGTTCGCTCTCGATAAC |
| pBAD-OsCOMT-F | GCTAACAGGAGGAATTAACCATGGGTTCTACGGCGGCG |
| pBAD-OsCOMT-R | CCGCCAAAACAGCCAAGCTTTTACTTGGTGAATTCAATAGCCCACGC |
| SaPsmH-F | GGTCGTCATCTGCGTTAATTTAACTTTAAGAAGGAGATATAATGAAGCCCGCTGACGCG |
| SaPsmH-R | CTACTCGGGCAGCGCATCAGCCGAGCCGCC |
| SaPsmF-F | GATGCGCTGCCCGAGTAGTTTAACTTTAAGAAGGAGATATAATGAACACCTTCCGGACC |
| SaPsmF-R | TCAGTCGCAGTGGTCATGGATCG |
| SaCOMT-F | ATGACCACTGCGACTGATTTAACTTTAAGAAGGAGATATAATGCCAGACGACCCGAGCC |
| OsCOMT-F | CATGACCACTGCGACTGATTTAACTTTAAGAAGGAGATATAATGGGTTCTACGGCGGCG |
| pZSSaCOMT-F | GACCACTGCGACTGATTTAACTTTAAGAAGGAGATATAATGCCAGACGACCCGAGCC |
| pZSSaCOMT-R | CCGCCAAAACAGCCAAGCTTTTAGACCGGTTCGCTCTCGATA |
| pZSOsCOMT-F | TGACCACTGCGACTGATTTAACTTTAAGAAGGAGATATAATGGGTTCTACGGCGGCG |
| pZSOsCOMT-R | CCGCCAAAACAGCCAAGCTTTTACTTGGTGAATTCAATAGCCCAC |
| FolM-R2 | TTAACGCAGATGACGACCGCCA |
| SaCOMT-F2 | CGTCATCTGCGTTAATTTAACTTTAAGAAGGAGATATAATGCCAGACGACCCGAGCC |
| OsCOMT-F2 | TCGTCATCTGCGTTAATTTAACTTTAAGAAGGAGATATAATGGGTTCTACGGCGGCG |
| pZH-F2 | CGATCCATGACCACTGCGACTGAAAGCTTGGCTGTTTTGGCGG |
| OsCOMT-F3 | CGCGACGGCGACGTGTAATTTAACTTTAAGAAGGAGATATAATGGGTTCTACGGCGGC |
| OsCOMTmutant-F | CGAGGGTATGAAAAACCATGCTGTGATCATCACCAAAAAAC |
| OsCOMTmutant-R | GTTTTTTGGTGATGATCACAGCATGGTTTTTCATACCCTCG |
| OsCOMTmutant-F2 | TTCCACGGTAGTTGACGTTGCTGGTGGTGTTGGCGCAAC |
| OsCOMTmutant-R2 | GTTGCGCCAACACCACCAGCAACGTCAACTACCGTGGAA |

Table S2. The OD_600_ of Melatonin Producing Strains in M9Y medium (n=3)

|  | 0h | 6h | 12h | 24h | 48h | 72h | 96h |
| --- | --- | --- | --- | --- | --- | --- | --- |
| EcMEL7 | 9.8±0.1 | 9.5±0.1 | 9.3±0.1 | 9.3±0.1 | 8.9±0.1 | 8.8±0.1 | 8.7±0.1 |
| EcMEL7+0.5%glycerol | 9.7±0.1 | 9.5±0.2 | 9.3 | 9.2±0.1 | 8.7 | 8.8 | 8.6 |
| EcMEL7+1.5%glycerol | 9.7±0.2 | 9.4 | 9.4±0.1 | 9.2±0.2 | 9.0±0.1 | 8.4±0.2 | 8.5±0.1 |
| EcMEL7+3%glycerol | 9.9±0.1 | 9.6±0.1 | 9.4±0.1 | 9.3±0.1 | 9.1±0.2 | 8.6±0.1 | 8.3±0.2 |
| EcMEL7+5%glycerol | 9.8±0.1 | 9.4±0.1 | 9.5 | 9.3±0.1 | 8.9±0.1 | 8.8 | 8.5±0.1 |


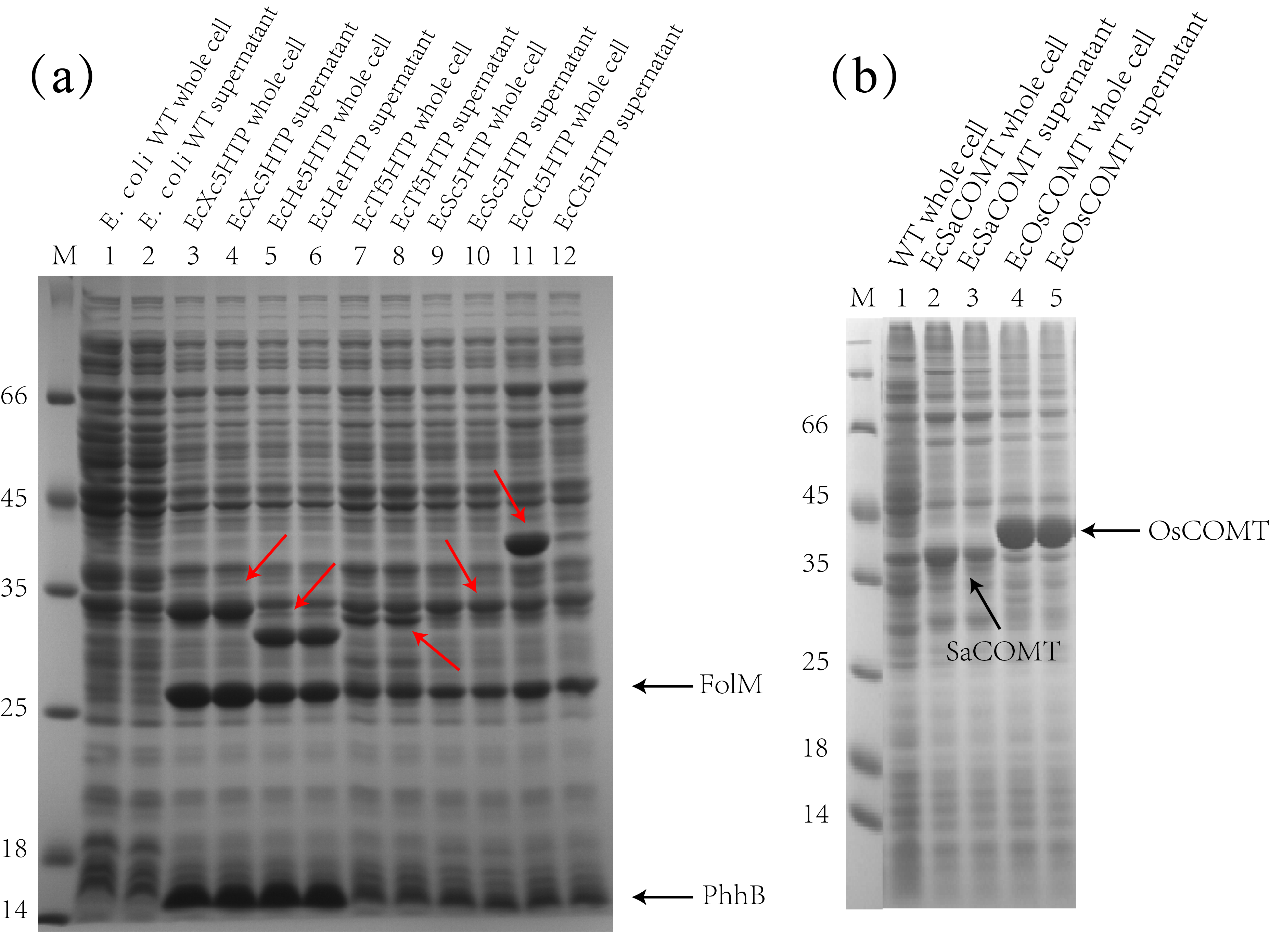


Fig. S1. (a), SDS-PAGE analysis of pBAD-5HTPs. The red arrow indicates the expression of P4Hs. (b), SDS-PAGE analysis of EcSaCOMT and EcOsCOMT


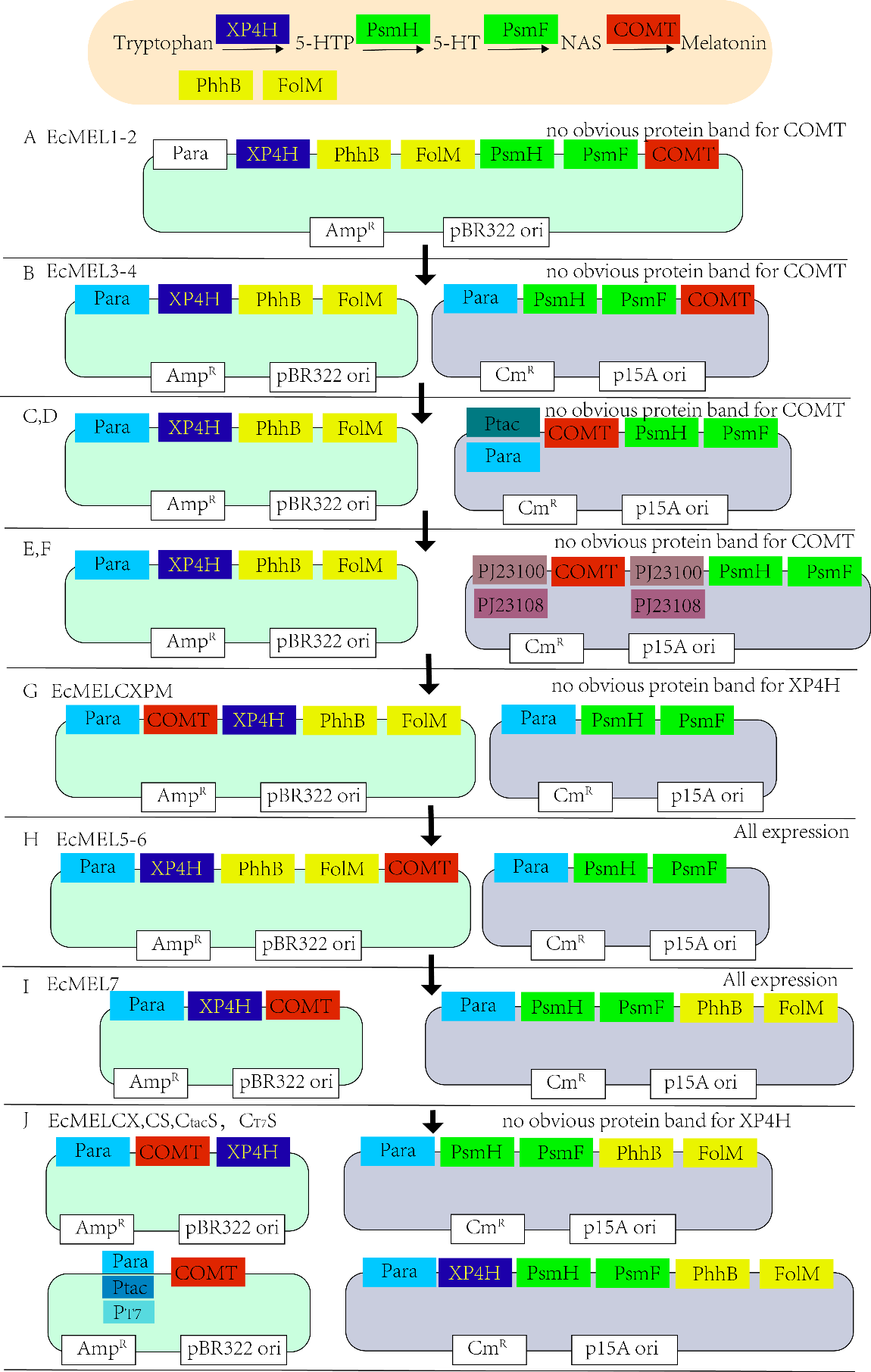


Fig. S2. Construction of plasmids for expression of melatonin-related proteins. A. pBAD-XP4H-PhhB-FolM-MH-MF-OsCOMT; pBAD-XP4H-PhhB-FolM-MH-MF-SaCOMT. B. pBAD-XP4H-Phhb-FolM/pZS-MH-MF-OsCOMT; pBAD-XP4H-Phhb-FolM/pZS-MH-MF-SaCOMT. C. pZS-SaCOMT -MH-MF (BW25113Δ*tnaA*)/ pZS-OsCOMT -MH-MF (BW25113Δ*tnaA*). D. pZS-Ptac-MH-MF-OsCOMT (BW25113Δ*tnaA*)/ pZS-Ptac-MH-MF-SaCOMT(BW25113Δ*tnaA*). E. pZS-PrJ23100-MH-MF-PrJ23100-OsCOMT (BW25113Δ*tnaA*)/pZS-PrJ23100-MH-MF-PrJ23100-SaCOMT (BW25113Δ*tnaA*). F. pZS-PrJ23108-MH-MF-PrJ23108-OsCOMT (BW25113Δ*tnaA*)/ pZS-PrJ23108-MH-MF-PrJ23108-SaCOMT (BW25113Δ*tnaA*). G. pBAD-OsCOMT-XP4H-PhhB-FolM. H. pBAD -XP4H-PhhB-FolM-OsCOMT/ pBAD -XP4H-PhhB-FolM-SaCOMT. I. pBAD-XP4H-OsCOMT. J. pBAD-OsCOMT-XP4H, pBAD-OsCOMT, pBAD-P_T7_-OsCOMT (BW25113(DE3) Δ*tnaA*), pBAD-P_tac_-OsCOMT.


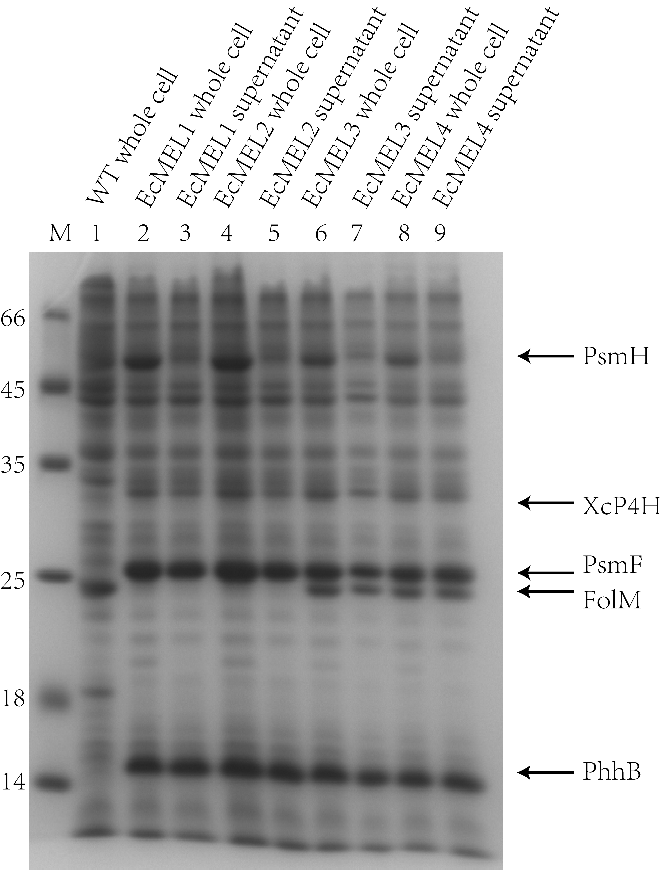


Fig S3. SDS-PAGE analysis of EcMEL1, EcMEL2, EcMEL3, and EcMEL4.


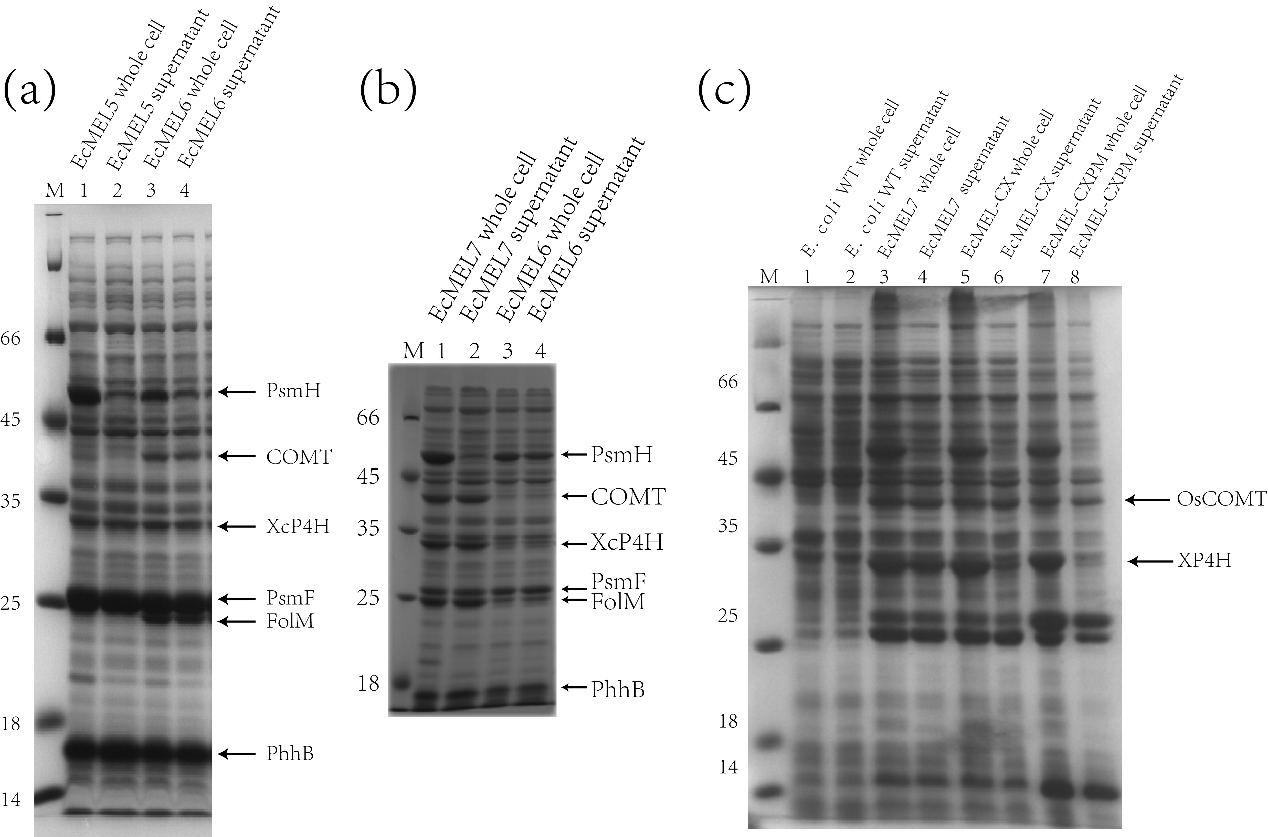


Fig S4. SDS-PAGE analysis of EcMEL5, EcMEL6, EcMEL7, EcMELCX and EcMELCXPM.


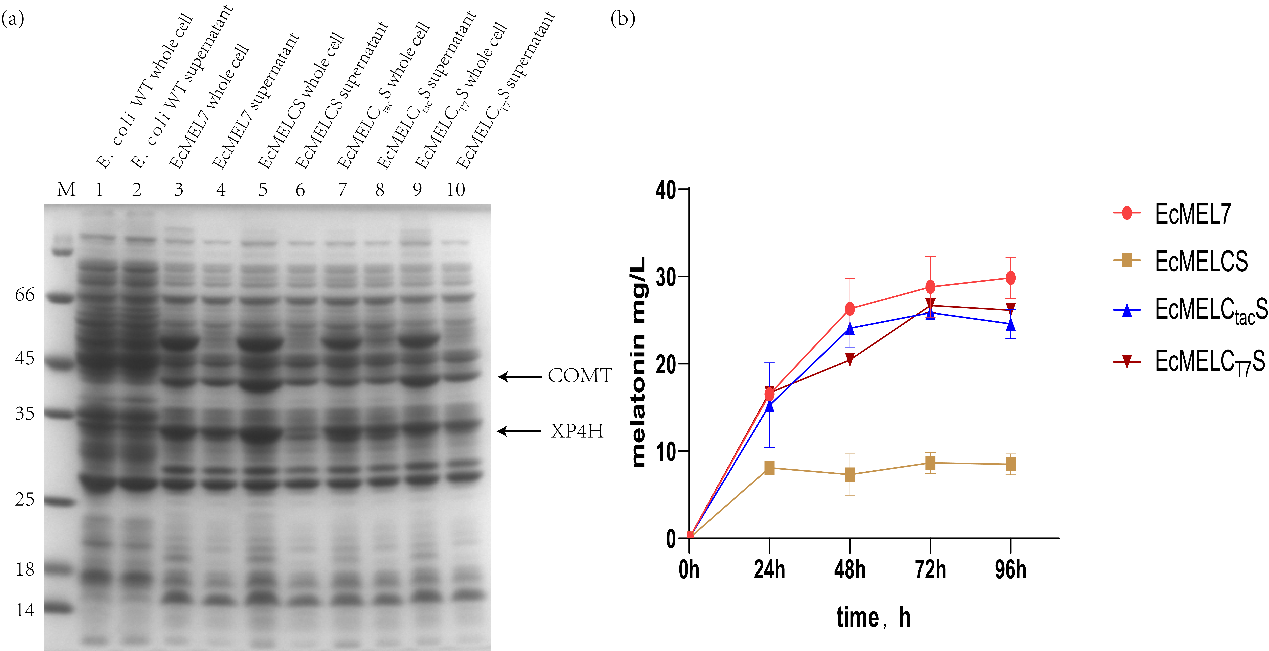


Fig. S5. Comparison of the melatonin production by EcMEL7,EcMEL-CS, EcMEL-CtacS and EcMEL-CT7S. (a) SDS-PAGE analysis of EcMEL7, EcMEL-CS, EcMEL-CtacS and EcMEL-CT7S. (b) Conversion of tryptophan to melatonin by recombinant E. coli strains.


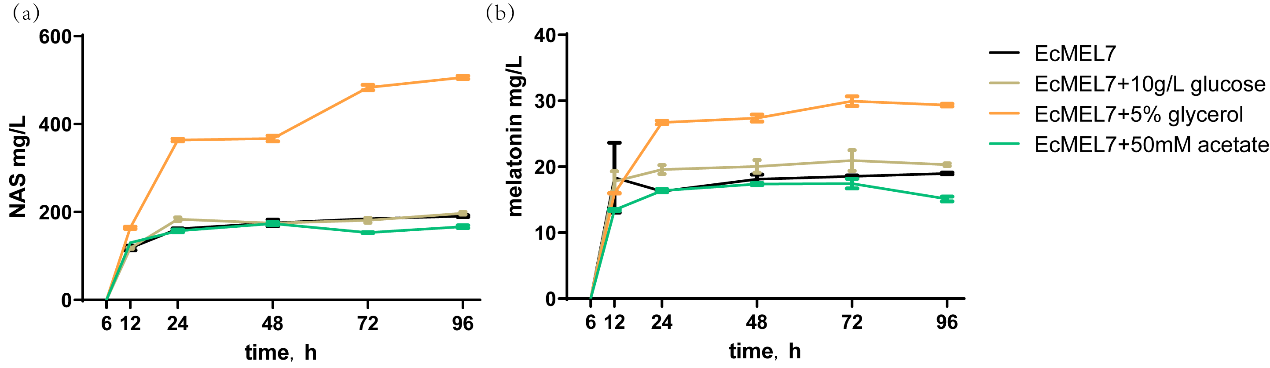


Fig S6. Comparison of adding glucose, acetate and glycerol in M9Y medium for biosynthesis of NAS and melatonin.


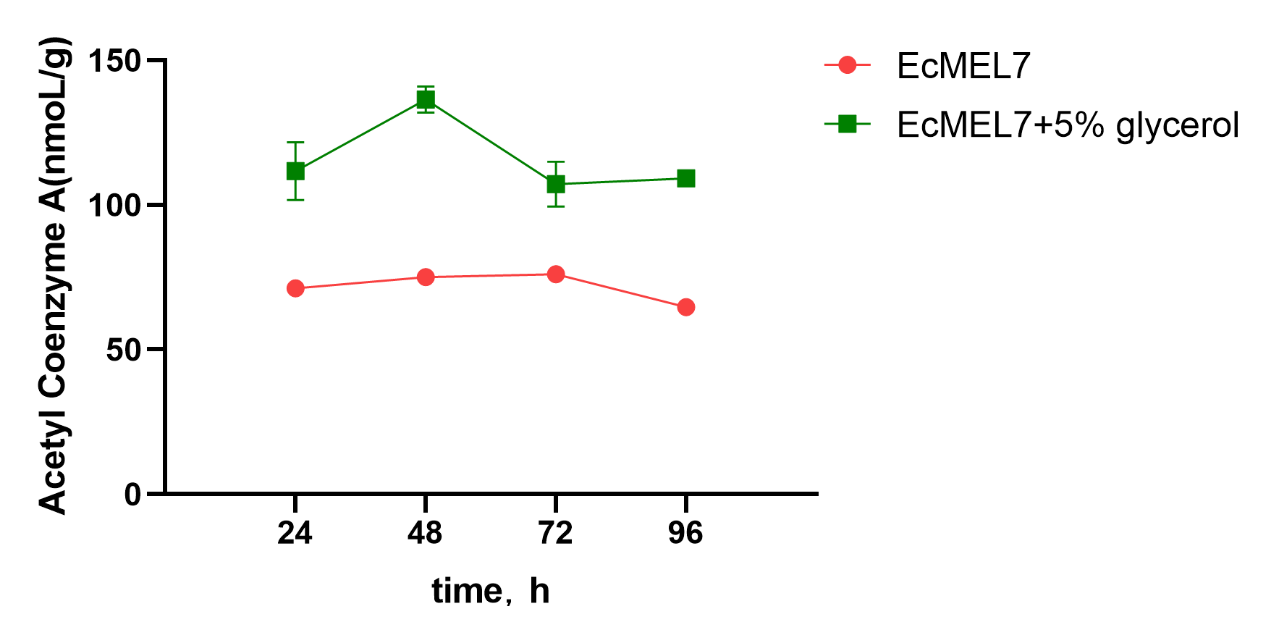


Fig. S7. The concentration of Acetyl Coenzyme A in EcMEL7 and EcMEL7+5% glycerol during the whole-cell biocatalysis. Data are the means ± standard deviations of triplicate experiments. The absence of error bars indicates that the error was smaller than the symbol size.


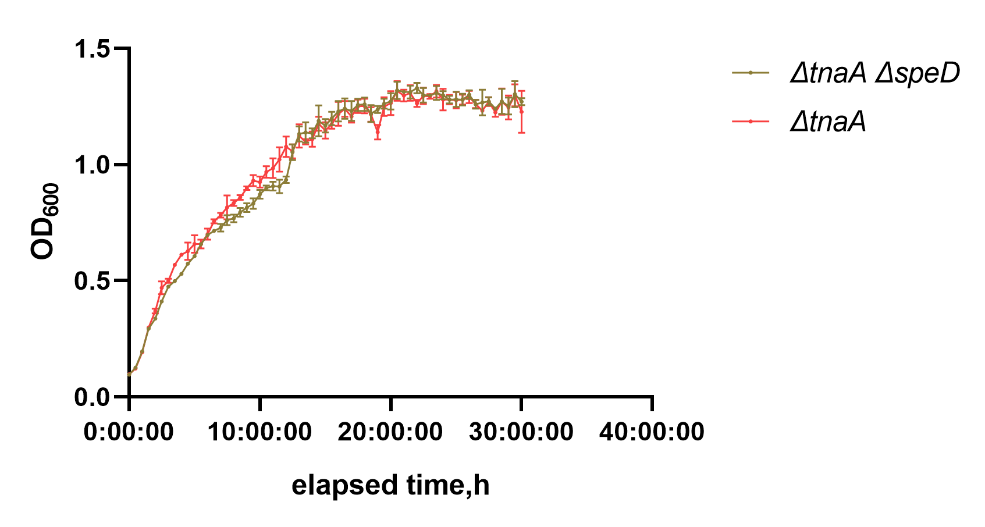


Fig. S8. The grow curve of BW25113Δ*tnaA* and BW25113Δ*tnaA* Δ*speD.* (n=3)

Table S3. The OD_600_ of Melatonin Producing Strains after adding glucose, glycerol, and acetate. (n=3)

|  | 0h | 24h | 48h | 72h | 96h |
| --- | --- | --- | --- | --- | --- |
| EcMEL7 | 10.0±0.1 | 9.4 | 9.0 | 8.7±0.1 | 8.4±0.2 |
| EcMEL7+10g/L glucose | 10.1±0.1 | 9.3±0.1 | 8.9±0.1 | 8.7 | 8.3±0.2 |
| EcMEL7+5%glycerol | 9.9 | 9.5±0.1 | 8.9±0.1 | 8.5 | 8.2±0.1 |
| EcMEL7+50mM acetate | 9.9±0.1 | 9.5±0.1 | 8.7±0.2 | 8.3±0.1 | 7.9±0.1 |
